# Hypothalamic MCH Neuron Activity Dynamics during Cataplexy of Narcolepsy

**DOI:** 10.1523/ENEURO.0017-20.2020

**Published:** 2020-04-29

**Authors:** Ying Sun, Meng Liu

**Affiliations:** Department of Psychiatry and Behavioral Sciences, Medical University of South Carolina, Charleston, SC 29425

**Keywords:** cataplexy, MCH, narcolepsy, sleep

## Abstract

Hypothalamic orexin (hypocretin, HCRT) deficiency causes sleep disorder narcolepsy with cataplexy in humans and murine. As another integral group of sleep/wake-regulating neurons in the same brain area, the melanin-concentrating hormone (MCH) neurons’ involvement in cataplexy remains ambiguous. Here we used the live animal deep-brain calcium (Ca^2+^) imaging tool to record MCH neuron dynamics during cataplexy by expressing calcium sensor GCaMP6s into genetically defined MCH neurons in orexin knock-out mice, which are a model of human narcolepsy. Similar to wild-type mice, MCH neurons of the narcoleptic mice displayed significantly higher Ca^2+^ transient fluorescent intensity during rapid eye movement (REM) sleep and active waking (AW) episodes compared with non-REM (NREM) sleep. Moreover, MCH neurons displayed significantly lower Ca^2+^ signals during cataplexy. Importantly, a pre-cataplexy elevation of Ca^2+^ signals from MCH neurons was not a prerequisite for cataplexy initiation. Our results demonstrated the inactivation status of MCH neurons during cataplexy and suggested that MCH neurons are not involved in the initiation and maintenance of cataplexy in orexin knock-out mice.

## Visual Abstract

**Figure:**
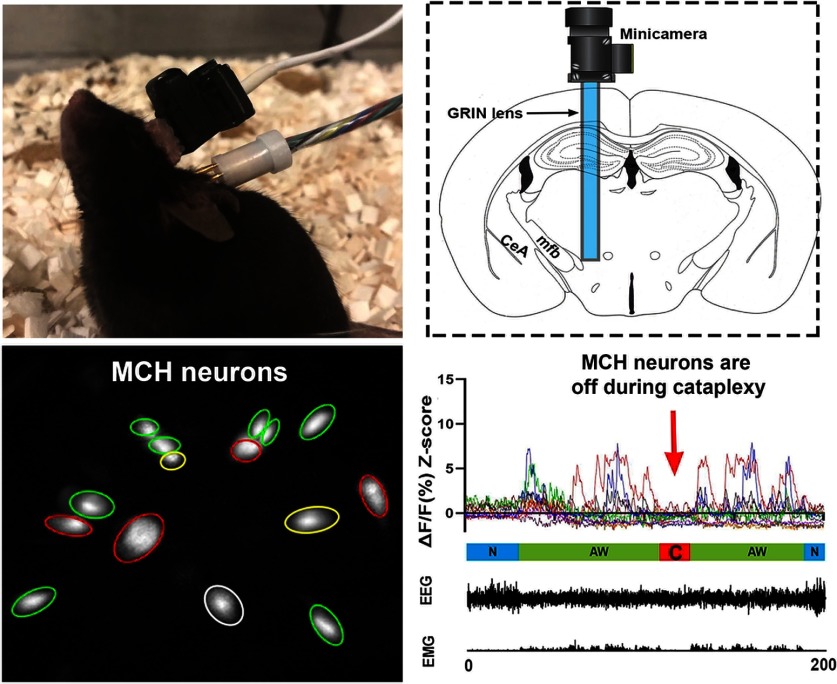

## Significance Statement

The cataplexy of narcolepsy shares many similarities with normal rapid eye movement (REM) sleep. Thus, REM sleep-promoting melanin-concentrating hormone (MCH) neurons are thought to be part of the cataplexy circuitry. Suppressing MCH neurons is assumed to block cataplexy. However, we found that MCH neurons were always inactive during cataplexy in orexin knock-out mice. Pre-cataplexy activations of MCH neurons were no different from the activations during regular waking episodes. Importantly, cataplexy could occur in the absence of these pre-cataplexy activations. These results suggest that MCH neurons are not a key component of the cataplexy circuitry in orexin knock-out mice.

## Introduction

Orexin (hypocretin, HCRT) and melanin-concentrating hormone (MCH) are hypothalamic neuropeptides regulating sleep and wakefulness. Orexin neurons are wake-active, promoting arousal and maintaining wakefulness (Adamantidis et al., 2007; Alexandre et al., 2013) while MCH neurons are predominantly rapid eye movement (REM) sleep-active and promote REM sleep (Jego et al., 2013; Konadhode et al., 2013; Blanco-Centurion et al., 2016, 2019; Izawa et al., 2019). Lack of orexin neurons or loss of the orexin gene causes sleep disorder narcolepsy and its signature symptom cataplexy, a sudden skeletal muscle atonia during waking (Chemelli et al., 1999; Lin et al., 1999; Hungs and Mignot, 2001). Cataplexy or cataplexy-like behavior has not been reported in MCH or its receptor knock-out mice, suggesting that there is no causal effect between MCH deficiency and cataplexy. However, whether MCH neurons play a role in cataplexy in narcolepsy patients or narcoleptic animals is still unknown. The question could be answered by manipulating MCH neurons in narcoleptic animals using optogenetic or chemogenetic tools. Indeed, a recent study indicated that chemogenetically activating MCH neurons increased cataplexy and abnormal REM sleep in orexin knock-out mice (Naganuma et al., 2018). However, we believe that the instinctive status of MCH neurons during cataplexy is still crucial for understanding the exact involvement of MCH neurons in cataplexy and for designing optimal intervention strategies to block cataplexy. For instance, if MCH neurons are active during cataplexy, further stimulation/excitation may have no noticeable effects due to the “ceiling” effect. Likewise, further inhibition might be ineffective due to the “floor” effect if MCH neurons are already silent during cataplexy. In this study, we took advantage of the novel genetic Ca^2+^ imaging tool to record real-time MCH neuronal activities during spontaneous cataplexy and emotional cataplexy induced by either positive (palatable food: milk) or negative (innate fear: predator odor) emotions. We want to know whether and how MCH neurons contribute to the generation or propagation of cataplexy.

## Materials and Methods

### Animals and surgery

All the manipulations done to the mice followed the policies established in the National Institutes of Health Guide for the Care and Use of Laboratory Animals and were approved by the Institutional Animal Care and Use Committee (protocol #IACUC-2019-00,723).

To specifically target MCH neurons in narcoleptic mice, orexin knock-out mice (*Hcrt*^−/−^) mice (derived from founders donated by Yanagisawa, Southwestern Medical Center, Dallas, TX) were crossed with MCH-Cre mice (The Jackson Laboratory; stock #014099). Offspring with the confirmed genotype *MCH-Cre*^+/−^*/Hcrt*^−/−^ were used as the narcoleptic group (*n* = 8, both sexes, 6–10 months of age). Genotype validation on mice tail snips was done off-site by Transnetyx. The temperature in the mice housing/recording room was always maintained at 23–25°C under a 12/12 h light/dark cycle (lights on at 6 A.M.). Mice were given *ad libitum* access to regular laboratory food and water.

Under deep anesthesia (isoflurane 1.0–2.0%) and using a stereotaxic frame (Kopf), adeno-associated virus (AAV) vectors with Cre-inducible expression of GCaMP6 slow (AAV5-CAG-DIO-GCaMP6s, Titer: 3.48 × 10^13^ genomic copies/ml; University of Pennsylvania Preclinical Vector Core) were microinjected unilaterally into the lateral hypothalamus at the following coordinates: 1.11 mm posterior to bregma, 1.25 mm lateral to the sagittal suture, and 4.60 mm ventral to the brain surface (Hof et al., 2000). Viral vectors were delivered in a volume of 500 nl using a 10.0-μl Hamilton syringe coupled to a 33-Gauge stainless steel injector (Plastics One). Injections were done gradually over 25 min. After microinjection, the injector needle was left in place for 15 min and then withdrawn slowly. At this time, and following the same injection track, a miniature Gradient Refractory INdex lens (GRIN, outer diameter: 0.6 mm, length: 7.3 mm; Inscopix Inc) was driven into the brain just above the injection target and cemented to the skull. Then, as described elsewhere (Liu et al., 2011), four small screw-type electrodes and a pair of plate-type electrodes (Plastics One) were implanted onto the mouse skull and nuchal muscles for recording the electroencephalogram (EEG) and electromyogram (EMG) activity, respectively. Ten days after the GRIN lens placement, mice were deeply anesthetized again (1.0–2.0% isofluorane). A baseplate was attached to a single photon miniaturized fluorescence microscope/CCD camera (nVoke from Inscopix). The miniaturized microscope, along with the baseplate, were carefully placed atop the GRIN lens. The distance between the miniaturized microscope and the GRIN lens top was precisely adjusted until fluorescent neurons came into focus. At this focal point, the baseplate was secured around the GRIN lens cuff with dental cement, and then the microscope was detached. To protect the GRIN lens from debris and scratches, a cap was secured onto the baseplate. One week later, mice were habituated to the recording experiment setting for three consecutive days before the sleep and Ca^2+^ recording started.

### Sleep recording and identification of sleep states or cataplexy

After being amplified and filtered (0.3–100 Hz for EEG; 100–1000 Hz for EMG, MP150 system; Biopac Systems Inc.), the EEG/EMG signals were acquired and synchronized to the imaging of the Ca^2+^ transients. In parallel, a night-vision camera was used to record mouse behavior. Streaming video of the mouse behavior was also synchronized with the imaging of the Ca^2+^ transients (Neuroscience Studio acquisition software, Doric Lenses Inc). NeuroExplorer software (Nex Technologies) was used to plot the spectrogram of the EEG activity (1-s window size and 0.5-s overlap).

The EEG/EMG data (as CSV files) along with synchronized behavior video files were then transferred to the SleepSign software (KISSEI Comtec Ltd.) and scored in 4-s epochs as wakefulness, non-REM (NREM) sleep, REM sleep, and cataplexy. Wakefulness was identified by the presence of desynchronized EEG coupled with high amplitude EMG activity. In this study, we focused on active wakefulness (AW) when the mice displayed behaviors such as walking, rearing, grooming, eating, drinking, digging, and exploring. NREM sleep was scored when the EEG showed high-amplitude/low-frequency waves (δ waves) together with a lower EMG activity relative to waking. REM sleep was identified by the presence of regular EEG θ activity coupled with very low EMG activity.

To be qualified as a cataplexy attack, an episode had to meet the following criteria: (1) an abrupt episode of nuchal atonia lasting at least 8 s, (2) immobility during the episode, (3) θ activity dominant EEG during the episode, and (4) at least 40 s of wakefulness preceding the episode (discrete cataplexy) or the first episode when several cataplexy episodes occur sequentially. The above criteria were slightly modified from the International Working Group on Rodent Models of Narcolepsy (Scammell et al., 2009). We also named the 40-s AW episodes preceding the cataplexy as pre-cataplexy AW episodes (Pre-C AW) to distinguish them from other AW episodes not followed by cataplexy (N-C AW).

### Miniature microscopy Ca^2+^ transients imaging

A t 10 A.M., the mouse was gently restrained (swaddled in Terrycloth), while a dummy miniscope (with the same size and weight as the real miniature fluorescent microscope) was attached to its baseplate. At the same time, a lightweight cable was plugged to record the EEG/EMG signals. The tethered mouse was then returned to the home cage and allowed to adapt for 6 h for three consecutive days. On the fourth day (the recording day), the same adaptation routine was followed with the real miniscope, but at 5 P.M., Ca^2+^ transients-derived fluorescence began to be imaged by the nVoke miniaturized microscope/CCD camera (Inscopix) and collected by its acquisition software. Ca^2+^ associated fluorescence was continuously generated by a blue LED (power: 0.2 mW) and imaged at five frames per second (fps). To synchronize the timestamps of the Ca^2+^ imaging with the EEG/EMG, a TTL signal was sent from the nVoke interface console into the Doric console. After 30 min of undisturbed recording, the mouse was exposed to milk (whole milk, 2 ml) for 30 min and coyote urine (1 ml, PredatorPee) for 30 min between 5:30 and 7 P.M. (Liu et al., 2016).

### Analysis of Ca^2+^ transients imaging data

The Ca^2+^ transient data were processed offline by the Inscopix data processing software (version 1.1.2). Briefly, raw movies were first pre-processed to correct for defective pixilation, row noise, and dropped frames. Preprocessed movies were then corrected for motion artifacts to generate the steadiest Ca^2+^ fluorescent signals. The motion-corrected movies were subsequently mean filtered. To normalize the Ca^2+^ signals, a single frame average projection of the filtered movie was generated. The average frame was used as the background fluorescence (F0) to calculate the instantaneous normalized Ca^2+^ fluorescent signals (ΔF/F) according to the formula; (ΔF/F)_i_ = F_i_ – F0/F0 where i represents each movie frame. The normalized movie or “ΔF/F movie” was then used for semiautomatic extraction of Ca^2+^ fluorescent signals associated with individual cells based on the principal and independent component analysis (PCA-ICA). Regions of interest (ROIs) identified by the PCA-ICA were visually selected as candidate cells based on ΔF/F and the image (cell-morphology). To be chosen as bona fide neurons, Ca^2+^ traces had to fulfill the canonic Ca^2+^ spike waveform featuring fast-rising onset followed by slower decaying signals. The Ca^2+^ traces (ΔF/F) of each ROI (cell) were further standardized as Z-scores using the mean and SD of each cell’s ΔF/F [Z-score = (ΔF/F – mean)/SD]. Since the lowest Z-score values in the normal sleep/wake states during the undisturbed recordings were observed in NREM sleep, and no cell reached its maximal activity during NREM sleep, we used the average Z-score of the NREM sleep (Z-_NREM_) as the baseline. If the average Z-score of a cell during a specific state is equal to or greater than (Z-_NREM_ +1.0) during the undisturbed recording period, it was scored as an “ON” cell in that state. We then used the percentage threshold, which was defined as 80% of the maximum Z-score value during the whole recording period, to detect the frequency of the prominent neuronal peak events (per cell, per minute), and we set 2 s as the minimum interval between two adjacent peaks.

### Histology

At the end of the study, the mice were anesthetized with isoflurane (5%) and perfused transcardially with 0.9% saline (5–10 ml) followed by 10% buffered formalin in 0.1 M PBS (50 ml). Mice brains were harvested and cross-sectioned at 40-μm thickness (four sets) on a compresstome (Precisionary Instruments). To visualize the GRIN lens track and the location of the GCaMP6s transgene expression, coronal sections were scanned on a Leica fluorescent microscope. To verify that GCaMP6s was expressed exclusively in MCH neurons, MCH immunostaining was performed on one set of brain sections. Briefly, the sections were incubated at room temperature for 24 h with rabbit anti-MCH polyclonal antibody (1:500 dilutions, Phoenix Pharmaceuticals), followed by 1-h incubation with Alexa Fluor 568 donkey anti-rabbit IgG (1:500). GCaMP6s and MCH-positive cells were counted on digitized images. To confirm the correctness of the genotyping, orexin immunostaining was conducted on a separate set of brain sections. Briefly, the sections were incubated at room temperature for 24 h with goat anti-orexin polyclonal antibody (1:5000 dilutions, Santa Cruz Biotechnology), followed by 1-h incubation with biotinylated donkey anti-goat IgG (1:500, Millipore) secondary antibody and finally labeled using ABC–DAB–nickel staining (Vector Laboratories).

### Statistical analysis

The one-way ANOVA and Bonferroni *post hoc* tests (SPSS, version 25) were used to compare the means of the Z-score values among the sleep/wake states. Statistical significance was evaluated at the *p* < 0.05 (two-tailed) level (Kirk, 1968).

## Results

### MCH/orexin immunostaining and GCaMP6s expression

**Figure 1.**
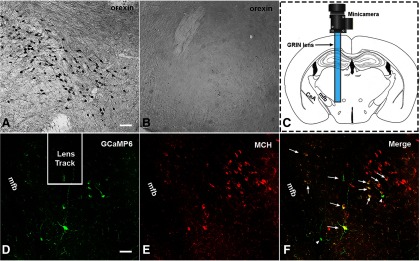
H istology results. Orexin^+^ neurons were found in the lateral hypothalamus of the wild-type MCH-Cre mice (***A***) but were absent in the MCH-Cre^+/−^/HCRT^−/−^ narcoleptic mice (***B***). ***C***, Illustration of the GRIN lens and miniature camera installation. ***D–F***, GCaMP6s and MCH expression in the hypothalamus near the end of the lens. Long arrows, GCaMP6s^+^/MCH^+^ double-labeled neurons. Short arrowhead, A GCaMP6s^+^/MCH^-^ neuron. CeA, the central nucleus of the amygdala; mfb, medial forebrain bundle. Scale bar = 50 μm. *Figure Contributions*: Ying Sun performed immunostaining and confocal imaging. Meng Liu produced the montage.

We found that orexin^+^ neurons were utterly absent in the *MCH-Cre*^+/−^*/Hcrt*^−/− ^mice used in this study, unlike the dense distributions of orexin^+^ neurons in the lateral hypothalamus of the wild-type MCH-Cre mice (Fig. 1*A*,*B*). These immunostaining results, together with the genotyping results and signature cataplexy behaviors, validated the animal model of narcolepsy. We recorded continuous Ca^2+^ transient signals from four of the eight mice installed with the GRIN lenses. In these four mice, GCaMP6s predominantly expressed within the lateral hypothalamus area (Fig. 1*C*,*D*). The MCH immunostaining results showed that ∼95–98% of the GCaMP6s expressing neurons also contained MCH in the cytoplasm (Fig. 1,*D–F*).

### MCH neuronal activities during undisturbed sleep/wake cycle

**Figure 2.**
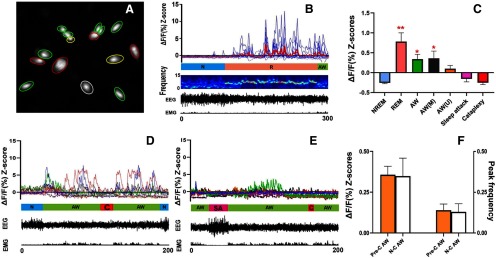
Ca^2+^ imaging/transient and synchronized behavioral data. ***A***, Map of 15 cells recorded from Mouse MLCH02. REM-ON cells are green circled, AW-ON cells are red circled, REM/AW-ON cells are yellow circled, and an unscored cell is white circled. ***B***, Plots of Ca^2+^ transient of the cells from ***A*** (thicker red line represents the average), followed by mouse EEG power spectrogram (1-s window size and 0.5-s overlap), EEG/EMG waveforms. N, NREM sleep; R, REM sleep; AW, AW. ***C***, Average Ca^2+^ transient signal intensity (ΔF/F Z-scores) of MCH neurons in various states. AW (*M*), AW during milk exposure; AW (*U*), AW during coyote urine exposure; **p < 0.001 or *p < 0.01 compared with NREM sleep, sleep attack, and cataplexy. ***D***, ***E***, Ca^2+^ transients and EEG/EMG waveforms from mouse MLCH03 after milk exposure (***D***) and predator odor exposure (***E***). C, cataplexy; SA, sleep attack. ***F***, No significant differences in Ca^2+^ signal intensities (Z-scores) and peak frequencies (peaks/cell/min) between Pre-C AW episodes and N-C AW episodes were found. *Figure Contributions*: Ying Sun and Meng Liu performed data analysis. Meng Liu produced the montage.

Altogether, we imaged 67 MCH neurons from four narcoleptic mice (two males and two females). These MCH neurons displayed activity patterns very similar to those of the wild-type MCH neurons (Blanco-Centurion et al., 2019; Fig. 2*A*,*B*; Movie 1). A marked increase in the average Z-scores was observed during REM sleep and AW when compared with NREM sleep (Fig. 2*C*). The results of the one-way ANOVA revealed a significant effect on the Ca^2+^ fluorescent intensity ΔF/F Z-scores (*F*_(6,468)_ = 58.38, *p* < 0.001) depending on individual sleep/wake states. The cells were categorized as ON (activated) cells based on the Ca^2+^ fluorescent intensity data before exposures to milk or coyote urine. Of the total 67 cells, 59.70% (40/67) were REM-ON only, 20.90% (14/67) were AW-ON only, 14.93% (10/67) were REM/AW-ON, and 4.48% (3/67) were unscored (no significant activity change among the different sleep/wake states was found during the undisturbed recording).

Movie 1. The raw calcium movies with synchronized EEG/EMG and animal behavior video during the undisturbed sleep recording shown in Figure 2*B* (played at 8x speed). 10.1523/ENEURO.0017-20.2020.video.1

### MCH neuronal activities during cataplexy and sleep attack

In addition to cataplexy, some narcoleptic mice may present another behavioral arrest called sleep attack, which is a brief NREM sleep intrusion during AW such as exploring, grooming, and eating. Sleep attacks were characterized by a mixture of δ and θ activities in the EEG, in contrast to cataplexy where θ activity dominates. We recorded 12 cataplexy bouts (three spontaneous ones before any exposure, four after the milk exposure, and five after the odor exposure) and eight sleep attack bouts (four before any exposure, two after the milk exposure, and two after the odor exposure) during the whole 2-h recording periods. The average Ca^2+^ intensity levels during cataplexy and sleep attack were similar to those of the NREM sleep and significantly lower than that of the REM sleep and AW. Importantly, neither “cataplexy-ON” nor “sleep attack-ON” cells were identified during the whole 2-h recording (Fig. 2*D*,*E*; Movie 2, Movie 3).

Movie 2. The raw calcium movies with synchronized EEG/EMG and animal behavior video during milk exploring and drinking shown in Figure 2*D* (played at 8x speed). 10.1523/ENEURO.0017-20.2020.video.2

Movie 3. The raw calcium movies with synchronized EEG/EMG and animal behavior video during predator odor exposure shown in Figure 2*E* (played at 8x speed). 10.1523/ENEURO.0017-20.2020.video.3

In the 30 min of undisturbed (before any exposure) and milk exposure recording, significantly elevated Ca^2+^ signal intensities from MCH neurons could be observed during N-C AW as well as AW episodes closely followed by a cataplexy bout (we named these episodes as Pre-C AW). The increased Ca^2+^ signal during these Pre-C AW suddenly dropped to the basal level once the cataplexy began (Fig. 2*D*,*E*). Upon the predator odor (coyote urine) exposure, the mice showed several typical behavioral signs of fear, including exploration, avoidance, and escape attempts. However, the Ca^2+^ signal intensities from MCH neurons showed insignificant increases during these fear response behaviors. In the meantime, sleep attack or cataplexy continued to be observed, during which time MCH neurons continued to display low Ca^2+^ fluorescent intensities (Fig. 2*E*; Movie 3).

To better understand the nature of the Ca^2+^ signal elevation during the pre-cataplexy episodes (except the AW episodes after predator odor exposure), we compared the Ca^2+^ signal intensities (average Z-scores) and peak frequencies between the Pre-C AW and the N-C AW, but we found no significant difference (Fig. 2*F*). Furthermore, we found that the sources or components of the Pre-C AW-ON cells and N-C AW-ON cells are very similar (Table 1). We did not find any significant difference between these two types of AW episodes.

**Table 1:** The numbers and percentages of on (activated) cells during the Pre-C AW were very similar to those during the N-C AW

	AW-ON only	REM-ON only	REMS-ON REMS/AW-ON	Unscored
N-C AW-ON cells (24)	14 (58.33%)	0 (0%)	10 (41.67%)	0 (0%)
Pre-C AW-ON cells (23)	12 (52.17%)	1 (4.35%)	9 (39.13%)	1 (4.35%)

No significant involvement of REM-ON only MCH neurons in Pre-C AW episodes was observed.

## Discussion

Cataplexy, presented as a sudden skeletal muscle paralysis or weakness during waking, is the most disabling symptom of sleep disorder narcolepsy (Reading, 2019). In murine models of narcolepsy, cataplexy usually occurs during the AW status of the animal, such as running, exploring, grooming, or responding to emotion stimuli (Overeem et al., 2001; Morawska et al., 2011; Reading, 2019). The brain circuitry responsible for cataplexy is not completely clear. Using the novel calcium imaging tool, we previously identified that the amygdala neuronal hyperactivity is associated with emotional cataplexy (Sun et al., 2019). In the present study, we recorded the *in vivo* activities of individual MCH neurons during cataplexy using the same methodology. We found that the majority of the recorded MCH neurons in the narcoleptic mice were most active during REM sleep, which is very similar to what occurs in wild-type mice (Blanco-Centurion et al., 2019; Izawa et al., 2019). Some recorded MCH neurons were also active during exploring, eating, and milk drinking, which is compatible with prior research about the effects of MCH neurons on food intake (Gonzalez et al., 2016; Dilsiz et al., 2020). Although prior studies indicated that MCH may regulate stress or fear (Carlini et al., 2006; Roy et al., 2006), we did not observe overt MCH neuron activation during the innate fear coyote urine exposure, implicating that the MCH neurons that were usually active during AW were suppressed by fear in the narcoleptic mice. Further research on wild-type mice MCH neurons is need to explore the exact correlation between MCH neurons and fear regulation.

Nevertheless, our results demonstrated that MCH neurons always stayed inactive during both spontaneous and emotional cataplexy (induced by milk or coyote urine), indicating that MCH neuron activation is not necessary for maintaining cataplexy in orexin knock-out mice. Although MCH neuron activation was observed in some pre-cataplexy episodes, the activation intensity, pattern, and ON cell components were very similar to the activation noted in the regular AW episodes. In contrast to the amygdala GABAergic neurons that displayed abnormal hyperactivity and increased activated cell numbers during the pre-cataplexy episodes that were followed by the coyote urine-induced cataplexy (Sun et al., 2019), neither hyperactivity nor recruitment of more REM-ON MCH cells has been found in these pre-cataplexy MCH neuron activations. This implicates that the pre-cataplexy activations are nothing different from the activations during regular AW. Noticeably, some cataplexy bouts could happen even when the MCH neuron activation was absent (Fig. 2*E*). All this evidence indicates that MCH neuron activation during the pre-cataplexy episodes might be unnecessary for or irrelevant to cataplexy triggering. A previous study reported activated MCH neurons during cataplexy by examining c-Fos and MCH double-labeled neurons (Oishi et al., 2013). However, the c-Fos expression is an indirect marker of neuronal activation and lacks temporal and spatial specificities. There is a possibility that c-Fos expression induced by the AW behaviors immediately before cataplexy is mistaken as having been induced by cataplexy. Our work provides the first direct evidence of phenotype-specific and cataplexy-specific neuronal activities associated with MCH neurons.

Cataplexy is the most disabling feature in narcolepsy patients. Because of the similarities of the EEG spectrum and muscle atonia features, cataplexy has long been considered as a REM sleep intrusion into waking or abnormal REM sleep during waking (Roth et al., 1969; Luppi et al., 2011). Indeed, studies have found that cataplexy and REM sleep share some common neural substrates (Thakkar et al., 1999; Dauvilliers et al., 2014; Peever and Fuller, 2016). Thus, MCH neurons, an important REM sleep-regulating hub, are thought to trigger intrusions of REM sleep bouts into waking in the absence of orexin, consequently resulting in cataplexy. In support of this hypothesis, chemogenetic activation of MCH neurons increased cataplexy and abnormal REM sleep (Naganuma et al., 2018). However, some emerging evidence is not supportive of this hypothesis. For instance, stimulating cholinoceptor in the basal forebrain of narcoleptic dogs induced cataplexy attacks but did not block the cyclicity of burst of REM, implying that separate mechanisms underlie cataplexy and REM sleep (Nishino et al., 2000). MCH neuron activation is highly correlated with REM sleep in normal mice, while we found an explicit disassociation between MCH activation and cataplexy. The above evidence suggests that cataplexy and REM sleep might have discrete mechanisms and MCH neurons stay outside the cataplexy circuit. However, it does not necessarily exclude the possibility that MCH activation triggers abnormal REM sleep and muscle weakness. In other words, the abnormal REM sleep intrusion or muscle weakness induced by stimulating MCH neurons exogenously is mechanistically distinct from, albeit similar to, the muscle atonia seen in cataplexy of narcolepsy. Moreover, the silence of the MCH neurons during cataplexy implicates that their appropriate activation may inhibit cataplexy. Indeed, a recent study demonstrated that ablating both orexin and MCH neurons worsens cataplexy, revealing the possible role of MCH neurons in preventing cataplexy (Hung et al., 2019).

Notably, unlike in the orexin neuron ablation models for human narcolepsy (Hara et al., 2001; Tabuchi et al., 2014), the original orexin neurons in the orexin knock-out mice are still intact despite the loss of orexin peptides. These neurons might affect the activity of MCH neurons via other neuropeptides coexisting with orexin, such as dynorphin (Chou et al., 2001). Thus, a similar calcium imaging study on the orexin neuron ablation model is needed to further confirm our findings.

Sleep attack occurs in some narcoleptic patients and mice. In humans, it takes place when an overwhelming sense of sleepiness comes on quickly and is considered a symbol of excessive daytime sleepiness. In mice, such an attack is manifested as a short NREM sleep intrusion into waking (Hishikawa et al., 1968; Liu et al., 2011). It is probably caused by the increased sleep drive when orexin is missing, or when orexin neurons die. The missing MCH neuron activation supports the NREM sleep mechanism of sleep attacks.

In conclusion, we found that MCH neuron activation is unnecessary for the maintenance and initiation of cataplexy in orexin knock-out mice. Thus, MCH neurons might not be one of the crucial nodes in the cataplexy brain circuitry in orexin knock-out mice.

## Synthesis

Reviewing Editor: Rae Silver, Columbia University Decisions are customarily a result of the Reviewing Editor and the peer reviewers coming together and discussing their recommendations until a consensus is reached. When revisions are invited, a fact-based synthesis statement explaining their decision and outlining what is needed to prepare a revision will be listed below. The following reviewer(s) agreed to reveal their identity: Mahesh Thakkar.

In this paper, the authors used calcium imaging tool to record MCH neuron dynamics during cataplexy in live mice. They found that MCH neurons were inactive during cataplexy, and that pre-cataplexy activations of MCH neurons were not different from the activation during regular waking episodes. Based on these observations, the authors concluded that MCH neurons were not involved in the initiation and maintenance of cataplexy in the orexin knockout mice.

Despite the rather low number of animals used in the experiments, presented results are clear. Furthermore, the paper is well constructed, the experiments were well conducted, and analysis was well performed. However, a few things could be added to the discussion. While the conclusion of the paper that MCH neurons were not involved in the initiation and maintenance of cataplexy is well supported by the results, the authors fell short in suggesting that MCH neurons normally suppress cataplexy, which seems to be very likely (https://www.biorxiv.org/content/10.1101/2019.12.17.880229v1.full). In addition, one potential caveat is that the absence of orexin in orexin knockout mice does not fully relate to the pathophysiology of narcolepsy in which orexin-producing cells are lost. These considerations were not mentioned in the paper.

Minor concerns.

Ca2+ transient signals were recorded in four out of the eight mice installed with the GRIN lenses. It is not clear why other four mice were not used. Is it because GCaMP6s expression was not sufficiently high in the lateral hypothalamic area in these mice? This needs to be clarified.

Did the authors see any differences in the results between male and female mice?

Would the results be the same if a cataplexy attack is defined as an abrupt episode of nuchal atonia lasting 10 or more seconds?

- The manuscript has several grammatical/typographical errors.

- There is no defined hypothesis. A statement with a defined hypothesis would be helpful.

- y-axis in all panels of Figure 2 should be labeled.
